# The Role of Brain-Derived Neurotrophic Factor in Epileptogenesis: an Update

**DOI:** 10.3389/fphar.2021.758232

**Published:** 2021-11-26

**Authors:** Xinyi Wang, Zhe Hu, Kai Zhong

**Affiliations:** Department of Pharmacology, School of Basic Medical Sciences and Forensic Medicine, Hangzhou Medical College, Hangzhou, China

**Keywords:** epilepsy, brain-derived neurotrophic factor, tyrosine kinase receptor B, treatment target, epileptogenesis

## Abstract

Epilepsy, which is characterized by spontaneous recurrent seizures, is one of the most common and serious chronic neurological diseases in the world. 30% patients failed to control seizures with multiple anti-seizure epileptic drugs, leading to serious outcomes. The pathogenesis of epilepsy is very complex and remains unclear. Brain-derived neurotrophic factor (BDNF), as a member of the neurotrophic factor family, is considered to play an important role in the survival, growth and differentiation of neurons during the development of the central nervous system. Recent years, a series of studies have reported that BDNF can maintain the function of the nervous system and promotes the regeneration of neurons after injury, which is believed to be closely related to epileptogenesis. However, two controversial views (BDNF inhibits or promotes epileptogenesis) still exist. Thus, this mini-review focuses on updating the new evidence of the role of BDNF in epileptogenesis and discussing the possibility of BDNF as an underlying target for the treatment of epilepsy.

## Introduction

Epilepsy, which currently afflicts approximately 0.5–1% of population in the world (Fisher et al., 2014; Chen L. et al., 2020), is a chronic progressive central nervous system (CNS) disorder with a sudden abnormal discharge of neurons in the brain. Epilepsy is characterized by spontaneous recurrent seizures (SRS), which can lead to a range of serious consequences including temporary brain dysfunction, accidental injury, neurological damage and cognitive decline (Thijs et al., 2019). The clinical treatment of epilepsy is mostly dependent on anti-seizure drugs (ASDs). However, about 30% patients still failed to control seizures after regular treatment with multiple ASDs, who may gradually develop into intractable epilepsy patients (Wang and Chen, 2019). So there is an urgent need for seeking new therapeutic drugs or strategies. Since the pathogenesis of epilepsy is not yet clarified clearly, more and more researchers focus on the mechanism research of epilepsy and expect to find new therapeutic targets *via* further understanding the underlying mechanisms of several complex neural circuits (Wang et al., 2017; Xu et al., 2019) and associated molecules (Wang and Chen, 2019; Xu et al., 2021).

Several important pathological processes, such as neuronal damage, regeneration, and abnormal neural circuits formation in the epileptogenesis, have been reported (Chen B. et al., 2020; Chen LY. et al., 2020). Accumulated studies have focused on some key molecules that may be involved in these pathological processes, including brain-derived neurotrophic factor (BDNF). BDNF is an important member of the neurotrophic factor family. It has a major role in promoting neuronal survival, growth and differentiation during the development of the CNS (Binder, 2004; Sharma et al., 2020); moreover, it can maintain the survival and function of the nervous system and promotes neuro-regeneration after injury (Song et al., 2008; Sun and Alkon, 2019). It was found that BDNF mainly exerts its effects through binding to at least two membrane receptors: tyrosine kinase receptor B (TrkB) and low affinity neurotrophic factor receptor (LNGFR, also known as p75) (Carito et al., 2014). TrkB is a high-affinity receptor for BDNF, while p75 is a low-affinity receptor, which is preferentially binded by BDNF precursor (proBDNF). A significant number of studies have shown an association between the BDNF-p75 pathway and psychiatric symptoms such as anxiety, depression and irritability (Lin and Huang, 2020), while very few studies have shown an effect of this pathway on epileptogenesis (Grabenstatter et al., 2014). The BDNF-TrkB pathway triggers a series of cascade reactions in downstream pathways, which is considered to play a main role in BDNF function (Naumenko et al., 2015). The signaling pathways downstream of the BDNF-TrkB include the following three: phospholipase Cγ, phosphatidylinositol 3-kinase and extracellular signal-regulated kinase cascade pathways, which are cross-linked to form a complex signaling network. All of them ultimately lead to the phosphorylation and activation of the cyclic adenosine monophosphate-response element-binding protein (CREB), which mediates the transcription of genes related to neuronal differentiation and survival (Cunha et al., 2010).

Basic and clinical studies have successively found that BDNF in the brain has a broad effect. It can alleviate neurological damage caused by diseases, such as Alzheimer’s disease, Parkinson disease, spinal cord injury, stroke, and other neurological disorders (de Boer et al., 2017; Tanila, 2017; Rosa et al., 2019; Vidal-Martinez et al., 2019), and is strongly associated with the development of cognitive function, learning, memory, and depression (Hing et al., 2018). For instance, BDNF couldregulate synaptic plasticity and long-term potentiation (LTP) in the hippocampus and other brain regions to affect learning and memory (Leal et al., 2014). Hippocampal LTP is damaged in mice lacking BDNF in their neurons, and BDNF can enhance synaptic plasticity and LTP in the hippocampus and visual cortex (Mattson, 2008). Due to synaptic plasticity and LTP being closely related with temporal lobe epilepsy (TLE) and long-term memory (Tramoni-Negre et al., 2017), it is speculated that BDNF may be involved in TLE and the long-term memory deficits after TLE (Hattiangady et al., 2020).

In recent years, BDNF and its downstream pathways have also been found to play a role in the epileptogenesis (Walczak et al., 2013), but its exact role and potential mechanisms have not been completely illuminated. Based on the available studies, two opposing hypotheses have been proposed, that is BDNF may facilitate or inhibit the process of epileptogenesis (McNamara and Scharfman, 2012; Lin et al., 2020).

Therefore, to further understand the role of BDNF in the epileptogenesis, we aim to review the two views by summarizing the relevant literature in recent years, which may provide certain references for further elucidating the formation process of epilepsy and finding new targets for epilepsy treatment.

## BDNF Expression Inhibits Epileptogenesis

The hypothesis suggested that the expression of BDNF can suppress epileptogenesis by a combined receptor-ligand pathway; and the TrkB binding to BDNF may be the most closely associated with epilepsy between its two corresponding receptor-ligand pathways (McNamara and Scharfman, 2012; Iughetti et al., 2018).

### Evidences From Preclinical Trials

In cell experiments, BDNF and proBDNF were found to have a strong regulation in neuronal injury and repair as well as plasticity of synapses, both of which are derived from a protein expressed in the CNS: pre-BDNF precursors (Pre-proBDNF). BDNF and proBDNF mainly bind to TrkB and p75, respectively (Walczak et al., 2013). Their binding activates a series of signal cascade responses that may suppress epilepsy by decreasing pyramidal neuron excitability (Gibon et al., 2015). It is well known that different processes of epileptogenesis eventually pass through two necessary pathways: increased excitability of excitatory synapses and diminished inhibition of GABAergic neurons (Pitkanen and Lukasiuk, 2009). So the results suggested that the proBDNF has another pathway except for transforming into BDNF, which also can suppress epilepsy. In an animal experiment, BDNF was also found to attenuate the decrease in the electrical potential of GABAergic neurons in the brain tissue of epilepsy model of rats (Cifelli et al., 2013). It is hypothesized that BDNF may inhibit epilepsy by promoting phosphorylation of certain subunits of GABAergic neurons to attenuate the disease-induced decrease in GABAergic neuronal excitability (Cifelli et al., 2013). In a recent study, loss of BDNF-TrkB signaling in the cortistatin-expressing interneurons resulted in behavioral hyperactivity and SRS in mice (Maynard et al., 2020). Meanwhile, BDNF could alleviate the inflammatory response associated with epileptogenesis and thus reduce the disruption of the blood-brain barrier, as well as reduce the number of SRS in an epilepsy model of rats (Bovolenta et al., 2010). These results also suggested that BDNF has an inhibitory effect on epileptogenesis. In addition, a study in epileptic pregnant rats and their offsprings reported that the treatment of phenytoin increased the level of BDNF in the offspring born by epileptic rats and exhibited neuroprotection effects. It is suggested that BDNF reduces the CNS damage caused by epilepsy through its neurotrophic effect and then reveals the inhibitory effect on the epileptogenesis (Soysal et al., 2016). Notably, the effects of drugs and hormones themselves on epileptogenesis could not be ignored in this study, so this study should be considered as a side or indirect evidence.

In the study of drugs, Chiu et al. investigated the protective effect of dexmedetomidine on brain injury in a rat model of kainic acid (KA) epilepsy and found that dexmedetomidine ameliorated KA-induced neuronal apoptosis in rats and increased the expression of BDNF and TrkB meanwhile (Chiu et al., 2019). Also by exploring the effect of pantoprazole on pentylenetetrazol-induced seizures in rats, it is found that pantoprazole treatment delayed seizures, protected memory, and likewise increased the level of BDNF in the brain (Taskiran et al., 2021). From the above two studies, it is hypothesized that BDNF expression may inhibit the formation and recurrence of epilepsy and improve the prognosis through the protective effect of neurons. In addition, it has been found that CREB is an important nuclear transcription factor that regulates signaling pathways in the brain and is closely related to the expression of its downstream BDNF proteins, forming the CREB/BDNF pathway (Jiang et al., 2021). A recent study showed that hesperidin can promote the activation of the CREB-BDNF pathway and maintain physiological levels of BDNF to inhibit pentylenetetrazol-induced convulsions in zebrafish (Sharma et al., 2020). Thus, these drug studies suggested that BDNF expression may inhibit epileptogenesis from a side as well.

In general, many cellular and animal studies have shown a consistent relationship between decreased levels of BDNF and increased incidence of epilepsy, or that recovery of BDNF expression may alleviate epilepsy in experimental epilepsy. Notably, we noted that many studies have found the importance of maintaining normal levels of BDNF in suppressing epileptogenesis, but more direct evidences are still needed to clarify whether increasing BDNF to above physiological levels can significantly suppress epileptogenesis. Furthermore, although these results have suggested that BDNF expression may play a role in inhibiting the epileptogenesis, the precise mechanisms remain unsolved. Therefore, it is worthy of further researching the exact molecular mechanism of epileptogenesis inhibited by BDNF and its corresponding pathways, which may mainly involve in the binding of BDNF and proBDNF to TrkB and p75 followed by a series of signaling cascade reactions, the regulatory role of BDNF on GABA, and the CREB/BDNF pathway and so on.

### Evidences From Clinical Studies

Only a few studies reported the relationship between serum levels of BDNF and epilepsy in patients. A clinical study in patients with TLE showed that BDNF serum level in patients with TLE was significantly lower than that in the healthy controlsand the BDNF serum levels correlated with epilepsy duration (Chen et al., 2016). Recently, Poniatowski et al.divided the subject patients (n = 143) into a generalized tonic-clonic seizure group (n = 50) and chronic epileptic patients (n = 93) to determine their serum BDNF levels after seizures separately compared to the control group (n = 48). They found a significant decrease in serum BDNF levels in patients after generalized tonic clonic seizures, at 1 and 72 h after seizures in this group (Poniatowski et al., 2021). These data suggest a direct relationship between the decrease in BDNF levels and the occurrence of epilepsy. However, it is not clear whether the decrease in BDNF levels causes the epileptogenesis or isonly a concomitant phenomenon of epilepsy. Hence, further clinical studies on this issue are warranted. In addition, BDNF is rarely reported worldwide as an epilepsy-related screening index, while BDNF-related genes are now found to be strongly associated with the development of epilepsy in patients. It has been found (Shen et al., 2016) that polymorphisms in BDNF-related genes (Val66Met) will lead to abnormal BDNF secretion and functional alterations, which in turn will affect the progressive course of the disease in patients with TLE. Other clinical studies have found that several miRNAs are involved in the BDNF-TrkB pathway and certain miRNAs can increase the expression of BDNF and activate its downstream signaling pathways, which have an inhibitory effect on the development of epilepsy (Wang et al., 2016). For example, it has been shown that the expression of CREB, a key molecule in the downstream signaling pathway of BDNF, and its corresponding genes increased at the original foci in patients with intractable epilepsy, while miR-124 may retard the process of epileptogenesis by reducing the expression of mRNA corresponding to CREB1 to diminish the effect of CREB1 on its downstream receptor NMDAR (Wang et al., 2016). The close relationship among miRNAs, the BDNF-TrkB pathway, and epilepsy suggests that miRNAs acting on the BDNF-TrkB pathway may be a promising antiepileptic treatment strategy.

## BDNF Expression Promotes Epileptogenesis

The other hypothesis suggests that abnormal activity or increased levels of BDNF play a positive role in inducing epilepsy and promoting epileptogenesis (McNamara and Scharfman, 2012; Iughetti et al., 2018).

### Evidences From Preclinical Trials

It has been reported that the deletion of TrkB in mice caused a significant reduction in behavioural evidence for epileptogenesis (He et al., 2004; Kotloski and McNamara, 2010), and BDNF and TrkB expression increased in the amygdala of rats with epilepsy and depression (Liu et al., 2013; Briz et al., 2015). Ghadiri et al. found that BDNF levels were reduced in the hippocampus of rats given 32 mg/kg progesterone in an epilepsy model, while the number of apoptotic cells in the ipsilateral hippocampus and the number of damaged neurons was significantly reduced. Thus, the data showed an antiepileptic effect, which suggested that BDNF may promote neuronal cell damage and apoptosis to induce epilepsy formation (Ghadiri et al., 2019). In addition, decreased percentage of BDNF alleles in KA-induced epilepsy model of rats causes stronger mossy fiber sprouting to lead to the formation of abnormal excitatory circuits in the brain, which are thought to be associated with the epileptogenesis (Skupien-Jaroszek et al., 2021). This finding is consistent with some previous studies that BDNF takes part in sprouting events in epilepsy (Danzer et al., 2002; Scharfman et al., 2002), which suggests BDNF may play a positive role for epileptogenesis *via* promoting the mossy fiber sprouting and the formation of abnormal circuits. In addition, BDNF induces ryanodine receptor channel-mediated Ca^2+^ release and reactive oxygen species (ROS) production (Yang et al., 2020), while oxidative damage to mitochondria leads to disruption of mitochondrial function and cell death signalling, allowing excessive ROS production and ultimately triggering epilepsy (Quan et al., 2020). Therefore, it is speculated that BDNF may induce epilepsy by causing an increase in ROS production. Overall, BDNF may contribute to the development of epilepsy by activating its downstream pathways to modulate a variety of factors, (e.g., neuronal cell damage and apoptosis, mossy fiber sprouting, increase in ROS, etc.).

TrkB is a main downstream molecule of BDNF. Reduced TrkB receptors in the amygdala and hippocampus can hinder the process of epileptogenesis in TrkB receptor-related knockout mice (Kotloski and McNamara, 2010). The mechanism is believed that increased TrkB receptor activity inhibits Cl^−^ efflux by reducing KCC2 expression, which inhibits GABAergic neuron function and induces epilepsy (Liu et al., 2014). Similarly, transient inhibition of the TrkB receptor by a TrkB inhibitor can effectively suppress TLE in mice (Liu et al., 2013). REST/NRSF, an upstream gene of BDNF, acts to prevent seizures by inhibiting the activation of the seizure-associated BDNF-TrkB pathway, suggesting a possible role of the BDNF-TrkB pathway in promoting epileptogenesis (Chmielewska et al., 2020). In addition, BDNF overexpression in astrocytes in a lithium-pilocarpine mouse model worsened their phenotype, while neuroprotective effects were exhibited after lithium-pilocarpine treatment in the mice with hippocampal neurons or astrocyte-specific genes deficient of TrkB and significant retention of spatial learning ability was observed in the mice with astrocyte-specific gene deletion of TrkB (Fernandez-Garcia et al., 2020). The data further suggests that the damaging effects of the BDNF-TrkB pathway on nerves may be associated with epileptogenesis.

In summary, these cellular and animal studies have shown that increased BDNF levels seem to be correlated with the incidence of epilepsy and increased BDNF may be companied with the neuronal cell damage. However, the related molecular mechanisms still require to be further investigated, including whether increased BDNF levels have a direct damaging effect on neuronal cells and whether high levels of BDNF can reverse their protective effect on neurons at low levels.

### Evidences From Clinical Studies

Although BDNF has a nutritional and supportive effect on neurons under physiological conditions, overexpression of BDNF seems to play an opposite role. Excessive BDNF significantly increases excitability of neurons and increases susceptibility to epilepsy, thus producing damage to neurons and possibly promoting epileptogenesis (Scharfman et al., 2002). BDNF was found to increase excitatory synaptic transmission and decrease the inhibitory effect of inhibitory neurotransmitters on activated synapses, thereby enhancing the transmission of epileptiform discharges in neural networks (Doherty et al., 2019). The clinical study also found that the majority of epileptic patients had higher levels of serum BDNF than healthy individuals, and the expression of BDNF and TrkB receptors were positively correlated. It is suggested that BDNF may play an important role in the occurrence and development of TLE through TrkB receptors (Iughetti et al., 2018). In addition, BDNF and its conjugated receptor (TrkB) is increased in both animal models and human epilepsy patients, especially in the temporal and hippocampal regions which provides a surgical strategy for epilepsy surgery. The distribution of BDNF showed the levels of BDNF and TrkB receptors in the cerebrospinal fluid of patients with TLE are higher than that in peripheral blood. This phenomenon was also seen in some drug trials and studies. Investigating 80 epilepsy patients, who were already using new ASDs, and 13 healthy subjects, a study found that BDNF levels were higher in patients with focal epilepsy and new ASDs levels were negatively correlated with BDNF levels in serum and positively correlated with total quality of life scores in patients with multiple new ASDs (Demir et al., 2020). Therefore, the serum or cerebrospinal fluid test of BDNF may be a convenient and reliable test for prognostic assessment of TLE and medications.

Excessive transcriptional expression of BDNF has also been investigated in epileptic patient (Martinez-Levy et al., 2018). The use of BDNF gene polymorphism to replace the corresponding amino acid (Val66Met replaced by methionine) has a good therapeutic effect on epilepsy, while inhibiting the secretion of activity-dependent BDNF can prevent seizures (Egan et al., 2003; Chen et al., 2004). Some epidemiological studies have also found that BDNF Met66 allele carriers are relatively less prone to epilepsy in patients with Rett syndrome (Nectoux et al., 2008), suggesting that altering the molecular structure of BDNF may have an inhibitory effect on epilepsy formation and BDNF may have an important role in promoting epilepsy formation. A recent investigation from Hong Kong and Malaysia for BDNF genotyping showed a significant correlation between BDNF and risk of symptomatic epilepsy in Malaysian Indians (Sha’ari et al., 2016), suggesting that BDNF polymorphisms may increase the incidence of epilepsy in Malaysian Indians. Thus, all these studies support the idea that high expression of BDNF in certain brain regions may cause epilepsy.

In the above clinical study, BDNF levels were indeed higher in the serum or the cerebrospinal fluid of epileptic patients than that in normal subjects. However, due to technical and ethical limitations, we are generally unable to measure the changes of BDNF in epileptic patients during the epileptogenesis. Therefore, we have not been able to determine whether this elevation of BDNF induces neuronal excitation to promote epilepsy, or it is simply a stressful increase of the body to repair neurons after the onset of epilepsy. More researches are needed to further elucidate this issue in the future.

### Outlook

Despite accumulated evidences have indicated BDNF is closely associated with epileptogenesis in recent years, the exact role of BDNF in the epileptogenesis is still controversial, which may be concerned with the expression level of BDNF, the brain region of expression, its upstream and downstream molecules and other factors. It is speculated that there exist two possible hypotheses needed to be further verified. One is the expression level of BDNF in the brain needs to be maintained in certain range; that means, too high or too low may be unbeneficial for preventing from epileptogenesis. The other is that the influence of BDNF expression on epileptogenesis in different neural circuits, brain area, or subgroup of neurons may also be diverse. In addition, its upstream and downstream molecules may be involved in epileptogenesis. However, the role of several molecules such as p75 on epileptogenesis is still unclear, which is worthy to be further studied and clarified. Therefore, more researches and more direct evidences are needed to further elucidate these issues in the future; especially the elucidation of the role of BDNF in different neural circuits, which may have important significance for further understanding of epileptogenesis. Furthermore, many proteins and molecules within the BDNF-related signaling pathway are expected to be molecular targets for clinical epilepsy detection and treatment, which may be exploited to participate in the clinical treatment, risk assessment and prognosis of epilepsy. Hence, BDNF-related translational research is also a research direction to be focused on in the future.

## Summary

BDNF is closely related to epilepsy, since its upstream related genes and downstream receptors are found to be involved in the epileptogenesis. Various proteins and molecules in the whole pathway cross each other to form a complex signal transduction network; meanwhile, it is regulated by various exogenous substances. It may play a key role in the excitatory/inhibitory balance of neurons, which also has important significance of the occurrence and development of epilepsy. Currently, despite BDNF showed its effect in several preclinical researches, clinical studies using BDNF as a therapeutic agent have not been encouraging. Thus, BDNF is considered as a potential therapeutic target but not a drug and the modulation of BDNF and its upstream or downstream molecules by other agents may have certain clinical feasibility.
